# The association between school bullying and internet addiction among adolescents: a moderated mediation model

**DOI:** 10.3389/fpubh.2025.1502726

**Published:** 2025-03-27

**Authors:** Lei Li, Jia Cai, Cong Wang, Yun-Fei Mu, Zhong-Yue Deng, Ai-Ping Deng, Hong-Jun Song, Xue-Hua Huang, Li Yin, Yi Huang, Jin Chen, Jun-Shu Zhao, Bing-Zhi Zhang, Hao Li, Mao-Sheng Ran

**Affiliations:** ^1^Mental Health Center, West China Hospital, Sichuan University, Chengdu, Sichuan, China; ^2^Institute of Psychiatry, West China Hospital, Sichuan University, Chengdu, Sichuan, China; ^3^West China School of Nursing, Sichuan University, Chengdu, Sichuan, China; ^4^Department of Clinical Epidemiology and Evidence-Based Medicine, West China Hospital, Sichuan University, Chengdu, Sichuan, China; ^5^Ya'an Fourth People's Hospital, Ya'an, Sichuan, China

**Keywords:** school bullying, internet addiction, stigma of mental illness, depression, anxiety

## Abstract

**Background:**

School bullying poses a serious threat to the mental well-being of adolescents. Although previous research has demonstrated a link between school bullying and internet addiction, the psychological mechanism remains poorly understood. This study aimed to explore the mediating roles of depressive and anxiety symptoms, as well as the moderating role of the stigma of mental illness.

**Methods:**

A cross-sectional survey among 82,873 middle and high school, college, and university students in Sichuan Province, China, was conducted for this study. Moderated mediation models were examined using PROCESS macros in SPSS 26.0.

**Results:**

The school bullying was positively correlated with internet addiction, with depression and anxiety symptoms partially mediating internet addiction, respectively. The stigma of mental illness significantly moderated this relationship, revealing a stronger association between school bullying, depression and anxiety symptoms, and internet addiction for adolescents with higher levels of stigma.

**Conclusions:**

These findings emphasize the importance of addressing depressive and anxiety symptoms as well as stigma of mental illness in interventions to prevent school bullying and internet addiction. Programs tailored to these factors are crucial for alleviating the negative impacts of school bullying on the mental health and online behaviors of adolescents.

## 1 Background

School bullying is a global phenomenon that threatens the health of adolescent students including the problems of verbal, physical, relational and cyberbullying (1). Extensive research has documented the detrimental effects of school bullying on adolescent development. Evidence from 28 western countries suggests that ~41% of adolescents may be victims of school bullying, which is strongly associated with psychological problem among adolescent students, such as, depression, anxiety, loneliness and sleep disorders (2–5). School bullying can lead to decreased academic performance and increased use of violence and substance abuse by victims and bullies (6–8). Internet addiction has also been considered one of those negative outcomes of school bullying, and the relationship between bullying and Internet addiction is firmly supported (9).

The incidence of Internet addiction is increasing, and there are gender differences (10, 11). Internet addiction can be defined as the uncontrolled internet use despite its negative impact (12, 13). Studies showed that Internet addiction has negative effects on well-being of adolescents, including psychological distress and decreased life satisfaction (14, 15). The self-medication hypothesis posits that individuals who have experienced stressful events tend to rely on substances and drugs to avoid a series of adverse consequences (16). Drawing on this framework, excessive internet use may function as a maladaptive coping strategy analogous to substance abuse, providing temporary relief from bullying-related distress (17). In other words, problematic internet usage may be associated with school bullying. When individuals are bullied by their peers, they may immerse themselves in online activities to avoid being bullied repeatedly and to cope with the negative effects of being bullied (18).

Victims of school bullying may perceive themselves in negative light, triggering not only a sense of sham but also low self-worth and feelings of depression (19–21). Ample evidence strongly supports the relationship between being bullied and adverse mental health outcomes such as depressive and anxiety symptoms (22–24). For example, individuals who have experienced bullying were closely associated with poor psychosocial outcomes, even after adjusting for the impact of initial psychosocial stress (23). A meta-analysis also showed that compared with those who have not experienced school bullying, victims who were bullied at the age of 8 were 1.94 times more likely to exhibit anxiety or depression symptoms at the age of 9 (25). Online social activities are a common coping strategy for individuals with anxiety and depression to relieve negative emotions (26). However, the psychological pathways linking school bullying to internet addiction remain insufficiently explored, particularly regarding potential moderating factors that may exacerbate this relationship. Further studies should be conducted to explore the relationship between school bullying and internet addiction.

Stigma of mental illness may exacerbate the psychological distress of victims of school bullying (27). Childhood abuse and victimization may be key factors influencing the perceived stigma of mental illness in patients (28). Evidence showed that stigma of mental illness among individuals with experiences of school bullying may exacerbate psychological distress (29–32). The stigma of mental illness may reduce self-esteem and is related to depressive and anxiety symptoms as well as deliberate self-harm (33, 34). Studies showed that individuals may use online activities (e.g., internet use) to cope with their psychological distress, such as stigma of mental illness (35, 36). The stigma of mental illness may intensify the relationship between school bullying and internet addiction. However, few studies have been conducted to identify the relationship among stigma of mental illness, school bullying, symptoms of depression and anxiety, and internet addiction.

Thus, this study aimed to explore the underlying mechanisms between school bullying and internet addiction, as well as the relationships among stigma of mental illness, school bullying, depression, anxiety and internet addiction in adolescents. In this study, we hypothesized that: First, school bullying may be positively associated with internet addiction; Second, the depression and anxiety symptoms may play mediating roles the relationship between school bullying and internet addiction, respectively; Third, the stigma of mental illness moderates the relationships among school bullying, depression, anxiety and internet addiction.

## 2 Method

### 2.1 Participants and procedure

We conducted a large-scale online survey among students in Sichuan province, China, from December 14th, 2022 to February 28th, 2023. The multi-stage cluster sampling procedure recruited 90,118 students from 162 educational institutions across Sichuan Province, including junior middle school, senior high school, and universities. Before completing the survey, we acquired all participants to provide online informed consent. Teachers and professors distributed the questionnaires to students via Quick Response (QR) code. This study was approved by the Ethics Committee of West China Hospital, Sichuan University (NO. 2022-1970).

### 2.2 Measures

#### 2.2.1 Sociodemographic characteristics

Sociodemographic information, including gender, age, ethnicity, grade, family income, and the only child status was collected. Detailed demographic information and related factors were described in our previous study (37).

#### 2.2.2 School bullying

School bullying was assessed by the Chinese version of the Program for International Student Assessment (PISA), a 6-item self-report questionnaire (e.g., “Other students left me out of things on purpose”). Responses were ranked on a 4-point scale (0 = never or almost never; 1 = several times a year; 2 = several times a month; 3 = Once a week or more). Total scores ranged from 0 to18. The PISA has been proven to be reliable and valid (38). In this study, the Cronbach's coefficient for the PISA was 0.918, indicating excellent internal consistency.

#### 2.2.3 Depressive symptoms

Depressive symptoms were evaluated by using the Chinese version of 9-item Patient Health Questionnaire (PHQ-9), which is a self-report questionnaire assessing the severity of depression. Each item was scored on a 4-point Likert scale (range: 0–3). The total scores range from 0 to 27. The PHQ-9 has been proven to be reliable and valid (39, 40). In this study, Cronbach's coefficient for the PHQ-9 was 0.948.

#### 2.2.4 Anxiety symptoms

The Chinese version of the Generalized Anxiety Disorder 7-item (GAD-7) scale was used to assess participants' anxiety symptoms. It is a self-report questionnaire for evaluating the severity of anxiety. Each item was scored on a 4-point Likert scale (range: 0–3). The total scores range from 0 to 21. The GDA-7 has been demonstrated to be reliable and valid (41, 42). In this study, Cronbach's coefficient of the GAD-7 was 0.968.

#### 2.2.5 Stigma of mental illness

Stigma of mental illness used to assess a adapted version of Link's Perceived Discrimination-Devaluation Scale (LPDDS) (43, 44). The LPDDS is a 13-item self-report questionnaire for assessing the severity of perceived stigma of mental illness. Responses were reported on a 4-point Likert scale, labeled “Strongly Agree” to “Strongly Disagree”. Scores were summed for each item (ranges: LPDDS = 13-52). This scale has good reliability in the general Chinese population (44). In this study, Cronbach's coefficient for the LPDDS was 0.926.

#### 2.2.6 Internet addiction

The 20-item Internet Addiction Test (IAT) (45), self-report questionnaire was used to assess Internet addiction. Each item was scored on a 5-point Likert scale (range: 1–5). The total scores ranged from 20 to 100, with a higher score indicating a higher level of internet addiction. The total score ≥40 was defined as internet addiction. The Chinese version of IAT has been proven to be reliable and valid (46). In this study, it had good internal consistency, with a Cronbach's alpha coefficient of 0.956.

### 2.3 Statistical analysis

Statistical analyses were performed using SPSS 26.0. Descriptive analysis was used to summarize the basic demographic characteristics of the study subjects. For categorical variables, frequencies (*n*) and percentages (%) were presented, while for continuous variables, mean and standard deviation (SD) were included. The relationships between school bullying, depressive symptoms, anxiety symptoms, Internet addiction, and the stigma of mental illness were initially examined using the Pearson correlation coefficient.

Based on the hypotheses, we conducted mediation and moderated mediation analyses using the SPSS PROCESS v3.5 software developed by Andrew F. Hayes (47). Specifically, PROCESS Model 4 was employed to test the mediation model, with school bullying as independent variable (X), depression and anxiety as mediating variables (M), respectively, and internet addiction as dependent variable (Y).

**Figure 1:**
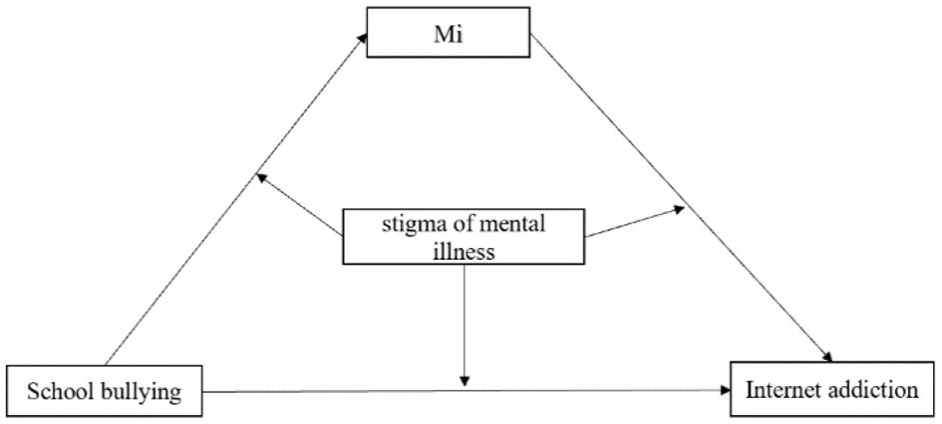
Hypothesized conceptual model of the moderated mediation. Mi represents mediating variables such as depression symptoms and anxiety symptoms.

We utilized PROCESS Model 59 to test the moderated mediation in the conceptual model (Figure 1) where stigma of mental illness served as the moderator variable (W). We defined two values for the stigma of mental illness: a low level (one standard deviation below the mean) and a high level (one standard deviation above the mean), to examine significant moderating effect. Additionally, simple slope computations were carried out for the moderation models to test the significance of the moderation slopes. To simulate the random sampling process and ensure the credibility of the study results, we used repeated sampling statistical method to verify the indirect effect of variable (48). This approach allowed us to estimate the variability of our results and provide a more accurate representation of the relationships among the study variables.

## 3 Results

### 3.1 Characteristics of the participants

In total, 82,873 students (92.0 %) provided informed consent and completed the questionnaire survey, while 7245 students (8.0 %) refused to participate. There were 47,487 female students (57.3%), 73,537 Han nationality students (88.7%), and 20,471 only-child students (24.7%). A total of 48,451 students (58.5%) had a monthly family income of less than 4,999 RMB. Table 1 shows the demographic characteristics of the participants in this study.

**Table 1:** Characteristics of the study participants.

**Variable**	***N =* 82873**
**Gender**, ***n*** **(%)**	
Male	35386 (42.7)
Female	47487 (57.3)
**Ethnic**, ***n*** **(%)**	
Han	73537 (88.7)
Ethnic minority	9336 (11.3)
**Grade**, ***n*** **(%)**	
Middle school	24157 (29.1)
High school	36111 (43.6)
College and university	22605 (27.3)
**Monthly family economy**, ***n*** **(%)**	
≤ 4,999	48451 (58.5)
5,000–19,999	30699 (37.0)
≥20,000	3723 (4.5)
**Only-child status** ***n*** **(%)**	
Yes	20471 (24.7)
No	62402 (75.3)
School bullying, mean (SD)	7.13 (2.89)
PHQ-9, mean (SD)	4.68 (6.03)
GAD-7, mean (SD)	3.39 (4.92)
IAT, mean (SD)	45.48 (16.99)
Stigma of mental illness, mean (SD)	32.26 (8.29)

IAT, Internet addiction test; PHQ-9, Patient health questionnaire-9; GAD-7, Generalized anxiety disorder-7.

### 3.2 Correlations between school bullying, depression, anxiety, internet addiction, and stigma of mental illness

Inter-correlations of all variables are presented in Table 2. Generally, school bullying was significantly positively associated with depressive symptoms (*r* = 0.409, *p* < 0.01), anxiety symptoms (*r* = 0.407, *p* < 0.01), and internet addiction (*r* = 0.261, *p* < 0.01). Depressive symptoms (*r* = 0.445, *p* < 0.01) and anxiety symptoms (*r* = 0.413, *p* < 0.01) were positively associated with internet addiction, respectively. Additionally, the stigma of mental illness was positively related to school bullying (*r* = 0.070, *p* < 0.01), depressive symptoms (*r* = 0.089, *p* < 0.01), anxiety symptoms (*r* = 0.089, *p* < 0.01), and internet addiction (*r* = 0.014, *p* < 0.01).

**Table 2:** Correlations between school bullying, internet addiction, depression and anxiety symptoms, and perceived stigma of mental illness.

	**School bullying**	**IAT**	**Depression (PHQ-9)**	**Anxiety (GAD-7)**	**Stigma of mental illness**
School bullying	1				
IAT	0.261^**^	1			
Depression (PHQ-9)	0.409^**^	0.445^**^	1		
Anxiety (GAD-7)	0.407^**^	0.413^**^	0.891^**^	1	
stigma of mental illness	0.070^**^	0.014^**^	0.089^**^	0.089^**^	1

IAT, Internet addiction test; PHQ-9, Patient health questionnaire-9; GAD-7, Generalized anxiety disorder-7; ^**^All *p*-values < 0.01.

### 3.3 Tests of mediation model

**Figure 2:**
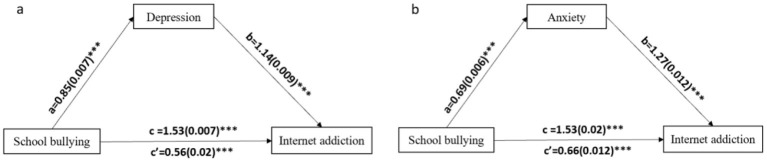
Path coefficients for the mediation model. **(a)** Mediating variable was depression, **(b)** mediating variable was anxiety. For each connecting line, the standard regression coefficient between those variables was shown. The 5,000-percentile bootstrapped standard error of each regression coefficient was shown in brackets. ***p < 0.0001.

Figure 2 showed the results of mediation analyses. As shown in Figure 2a, school bullying was positively associated with depression (β = 0.85, *p* < 0.0001), which in turn was positively associated with internet addiction (β = 1.14, *p* < 0.0001). The direct relationship between school bullying and internet addiction was also significant (β = 1.53, *p* < 0.0001), indicating that depression partially mediated the linking between school bullying and internet addiction (Supplementary Table 1). The indirect effect between school bullying and internet addiction was also significant (indirect effect = 0.97, 95% CI = 0.95–1.00, which accounted for 64.3% of the total effect) (Supplementary Table 2).

In additional, Figure 2b showed that school bullying was positively associated with anxiety (β = 0.69, *p* < 0.0001), which in turn was positively associated with internet addiction (β = 1.27, *p* < 0.0001). The direct relationship between school bullying and internet addiction was also significant (β = 1.53, *p* < 0.0001), indicating that anxiety partially mediate the linking between school bullying and internet addiction (Supplementary Table 3). The indirect effect between school bullying and internet addiction was significant (indirect effect = 0.88, 95% CI = 0.85-0.90, which accounted for 57.5% of the total effect) (Supplementary Table 4).

### 3.4 Tests of moderated mediation model

**Figure 3:**
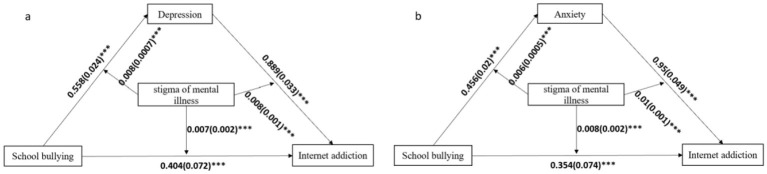
Path coefficients for the moderated mediation model. **(a)** Mediating variable was depression, **(b)** mediating variable was anxiety. For each connecting line, the standard regression coefficient between those variables was shown. The 5,000-percentile bootstrapped standard error of each regression coefficient was shown in brackets. ***p < 0.0001.

Stigma of mental illness can act as an “amplifier” to significantly enhance the negative effects between school bullying, depression, anxiety and internet addiction. As shown in Figure 3, the results of the moderated mediation analyses. In the Figure 3a, firstly, the stigma of mental illness moderated the mediating effect of depression between school bullying and internet addiction, and the interaction effect of school bullying and the stigma of mental illness on internet addiction was significant (β = 0.007, *p* < 0.0001), which indicates that the stigma of mental illness moderated the direct path between school bullying and Internet addiction. Secondly, the interaction effect of school bullying and the stigma of mental illness on depression was significant (β = 0.008, *p* < 0.0001), suggesting that the stigma of mental illness moderated the indirect path between school bullying and depression. Thirdly, the interaction effect of depression and the stigma of mental illness on internet addiction was significant (β = 0.008, *p* < 0.0001), indicating that the indirect path between depression and internet addiction was moderated by the stigma of mental illness (Supplementary Table 5).

In the Figure 3b, the stigma of mental illness moderated the mediating effect of anxiety between school bullying and internet addiction, and the interaction effect of school bullying and stigma of mental illness on internet addiction was significant (β = 0.008, *p* < 0.0001). The stigma of mental illness moderated the direct path between school bullying and Internet addiction. Secondly, the interaction effect of school bullying and the stigma of mental illness on anxiety was significant (β = 0.006, *p* < 0.0001), indicating that the stigma of mental illness might moderate the indirect path between school bullying and anxiety. Thirdly, the interaction effect of anxiety and stigma of mental illness on internet addiction was significant (β = 0.010, *p* < 0.0001), indicating that the indirect path between anxiety and internet addiction was moderated by the stigma of mental illness (Supplementary Table 6).

### 3.5 Simple slope analysis

**Figure 4:**
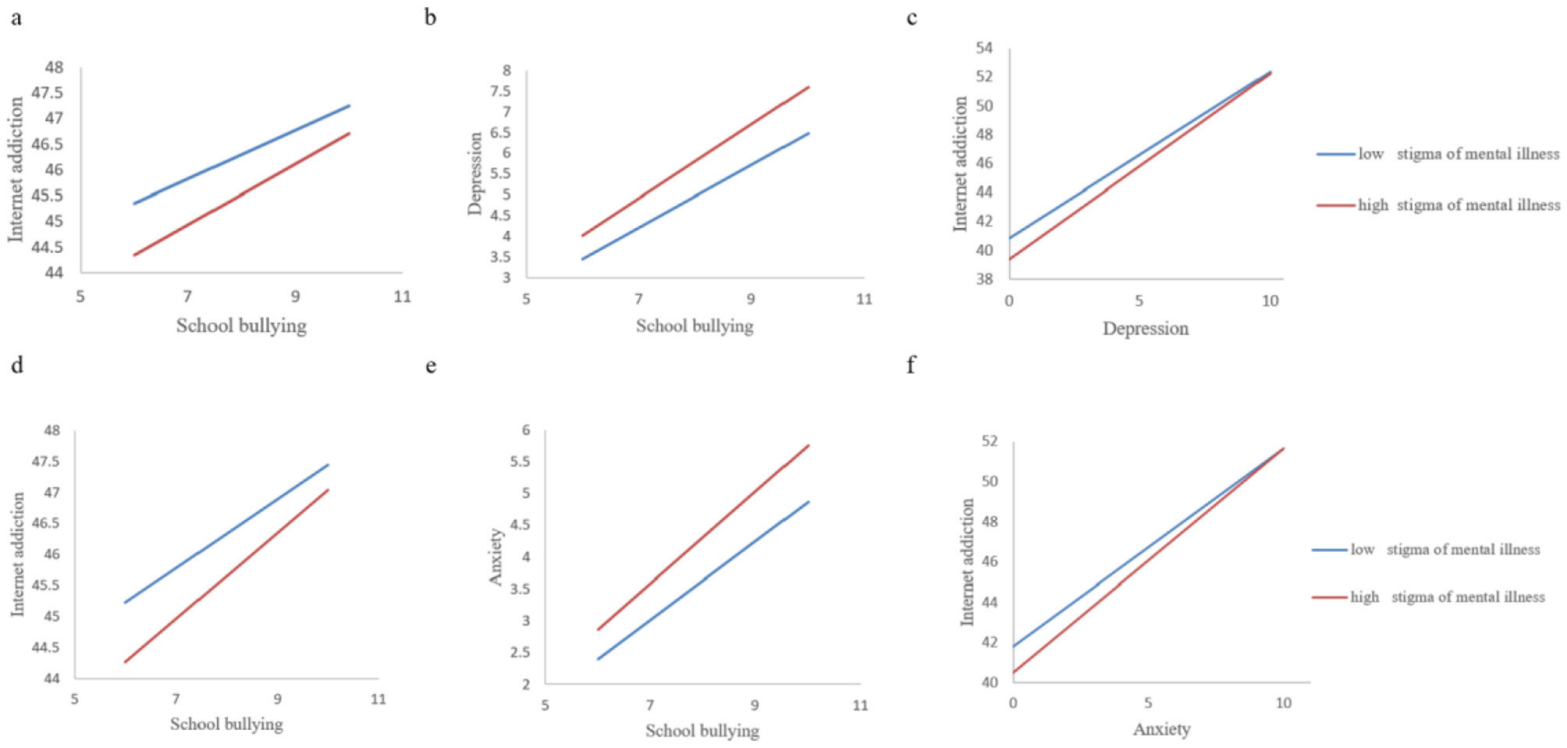
Moderation of the effect of stigma of mental illness between school bullying on internet addiction. **(a)** Stigma of mental illness moderated the relationship between school bullying and internet addiction. **(b)** Stigma of mental illness moderated the relationship between school bullying and depression. **(c)** Stigma of mental illness moderated the relationship between depression and internet addiction. **(d)** Stigma of mental illness moderated the relationship between school bullying and internet addiction. **(e)** Stigma of mental illness moderated the relationship between school bullying and anxiety. **(f)** Stigma of mental illness moderated the relationship between anxiety and internet addiction.

The Figure 4 showed that, regardless of whether a simple slope analysis was conducted for the low or high stigma levels of mental illness, school bullying had a significant positive impact on Internet addiction. Under the low stigma level of mental illness, the effect was smaller, indicating that as the stigma of mental illness decreased, the likelihood of Internet addiction when being bullied also decreased (Figures 4a, d). Secondly, at both high and low levels of stigma, the indirect effects of school bullying on Internet addiction through depressive and anxiety symptoms were statistically significant, and the indirect effects gradually decreased as the stigma level decreased. This showed that as the stigma of mental illness decreases, the impact of school bullying on depressive and anxiety symptoms can be effectively alleviated (Figures 4b, e). Finally, depressive symptoms had a significant positive impact on Internet addiction in the high stigma group, and depressive symptoms also had a significant positive impact on Internet addiction in the low stigma group (Figure 4c). Similarly, in the high stigma mental illness group, the impact of anxiety symptoms on Internet addiction was significantly higher than that in the low stigma mental illness group (Figure 4f).

## 4 Discussion

This study extends prior research by systematically examining how stigma of mental illness moderates the mediated pathways linking school bullying to internet addiction through depression and anxiety—a novel contribution to the literature. Our results fully confirmed the first and second hypotheses, demonstrating that school bullying is linked to internet addiction, and that symptoms of depression and anxiety mediate this association. Furthermore, we found that the stigma of mental illness moderates the association among school bullying, depression and anxiety symptoms, and internet addiction, which fully supports the third hypothesis. Specifically, although there is a strong relationship among school bullying, depression and anxiety symptoms as well as internet addiction, the linkage between those variables were stronger when the level of stigma of mental illness was high.

### 4.1 The association between school bullying and internet addiction

The results of this study demonstrate the link between school bullying and internet addiction, consistent with previous studies (49). Adolescents who experiencing bullying may be more likely to develop internet addiction as a coping mechanism, using online using online platforms to manage negative emotions, establish virtual relationships, and compensate for a lack of social connections in the real world. These behaviors may provide a sense of belonging and escape from the distress caused by bullying (50). Bullying has become one of the causes for the increase in psychological and medical problems. The necessity to prevent bullying incidents from recurring and to stop bullies from further harassing the victims has grown (51). These findings underscore the importance of developing psychosocial services and interventions to reduce school bullying and prevent internet addiction among adolescents.

### 4.2 Mediating role of depression and anxiety symptoms between school bullying and internet addiction

The mediating effect analysis revealed that symptoms of depressive and anxiety play pivotal roles in the relationship between school bullying and internet addiction. Specifically, school bullying increases the risk of depression and anxiety symptoms, and these symptoms, in turn, increase the risk of Internet addiction. This finding is consistent with previous research, which indicates a strong association between school bullying and mental health problems (2, 52, 53).

Adolescents experiencing bullying in the early school years reported negative emotions, such as depression or anxiety. They are often associated with the bullying incidents (54). Several mechanisms may underline the relationships between school bullying and mental health problems. Firstly, being bullied disrupts emotional regulation, leading to an increase in negative emotions and impaired emotion management, thus contributing to the occurrence of internalized anxiety and depression problems (55). Secondly, being bullying fosters rumination. Through continuous negative thoughts and fears about the bullying experience, rumination can lead to an increase in depressive and anxiety symptoms (56). Thirdly, bulling can affect adulthood through a stress process model. The traumatic stress caused by bullying can alter stress responses, leading to a long-term increase in inflammatory processes, overwhelming the psychological and biological stress processes of the victims (57).

Depressive and anxiety symptoms can have numerous negative consequences, including sleep disturbance, and internet addiction (58, 59). Students who have experienced school bullying may prefer to communicate with others through social media platforms, sharing their bullying experiences and seeking comfort, rather than interacting with familiar individuals in the real world, particularly when they feel an increased sense of loneliness (60). These findings emphasize the need for psychosocial interventions to reduce internet addiction, depression and anxiety symptoms, and improve the mental health outcomes among students who have experienced school bullying. By addressing the underlying mechanisms and consequences of school bullying, interventions can be tailored to meet the specific needs of this vulnerable population.

### 4.3 Stigma of mental illness moderated the mediated relationships

A key finding of this study was that the stigma of mental illness plays a moderating role in the relationship between school bullying, depression and anxiety symptoms, and internet addiction among adolescent students. It is noteworthy that both high and low levels of stigma of mental illness were positively correlated with anxiety, depression and Internet addiction. However, the positive association with mental illness was stronger at the high level of stigma, suggesting that a high level of stigma may exacerbate the relationships between school bullying, mental health problems, and internet addiction. This finding is consistent with existing research which demonstrates a link between the stigma of mental illness and various forms of psychological distress (30, 31). An intensified stigma associated with mental illness may lead to increased vulnerability to negative psychological outcomes, such as depression and anxiety, among these who have experienced school bullying. In addition, a high level of stigma may also contribute to increased online activities and decreased help-seeking behaviors, including reluctance to receive psychological or pharmacological treatment (61).

The results of this study underscore the critical role of stigma of mental illness in the complex relationships among school bullying, depression and anxiety, and internet addiction. The differential moderating effects of stigma suggest that interventions should adopt a tiered approach: universal anti-bullying programs for all students, combined with targeted stigma-reduction strategies for high-risk subgroups exhibiting elevated psychological distress (62). By addressing the moderating effect of stigma, interventions can be designed to more effectively mitigate the negative consequences of school bullying and promote mental health and well-being among students (63).

In this study, we utilized the LPDDS to assess stigma of mental illness, which specifically measures perceived stigma rather than internal stigma. It is noteworthy that victims of bullying often experience severe emotional distress due to psychological and physical violence, as well as social marginalization among their peers (64). This can lead to the development of a negative self-concept, decreased self-esteem, and perceived weakness (65). Research showed that individuals who perceive a higher degree of stigma are more likely to internalize and self-stigmatize (66). Both perceived and self-stigma can have deleterious effects on students' psychological well-being, treatment-seeking behaviors, and academic performance (67).

This study has significant implications for clinical practice. Firstly, it highlights the importance of considering experiences of school bullying as well as depression and anxiety symptoms when addressing students' internet addiction. The implementation of anti-bullying programs and intervention techniques has been shown to be effective in reducing bullying and victimization (68, 69). Given the crucial role that depressive and anxiety symptoms play in linking school bullying to internet addiction, it is essential to take actions to help students manage these symptoms and enhance treatment outcomes.

## 5 Limitations

This study has several limitations that should be acknowledged. As a cross-sectional study, we are unable to determine the causal relationships among variables. Future research could adopt a longitudinal design or an experimental design to further validate these relationships. While the large sample size enhances statistical power, the regional focus on Sichuan Province—an area with specific socioeconomic and educational characteristics—may limit the generalizability of findings to other cultural contexts. Future studies could be conducted in other regions or countries to verify whether our findings are widely applicable. Although we employed validated scales to minimize measurement error, the cross-sectional design precludes causal inferences, and self-reports may be influenced by recall bias or underreporting of stigmatized experiences. We believe that the large sample size, the validated scales, and the innovative introduction of 'stigma of mental illness' as a moderating variable have provided a solid theoretical foundation and empirical support for our research. We look forward to future research that further explores these complex relationships and employs multiple data collection methods to enhance the reliability of the research results.

## 6 Conclusion

This study contributes to understanding the associative mechanisms between school bullying and internet addiction. It explores the mediating roles of depressive and anxiety symptoms, as well as the moderating role of the stigma of mental illness. Depressive symptoms and anxiety symptoms play mediating roles between school bullying and internet addiction, respectively. Notably, the mediating effects of both depressive and anxiety symptoms were stronger for among students who report a higher level of stigma related to mental illness. These results point out that when formulating and implementing effective interventions for students who have suffered from school bullying, there is an urgent need to address depressive and anxiety symptoms and reduce the stigmatization of mental health.

## Data Availability

The data analyzed in this study is subject to the following licenses/restrictions: The de-identified data are available on reasonable request to the corresponding author. Requests to access these datasets should be directed to msrancd@outlook.com.
