# Supplementary material for: The association between school bullying and internet addiction among adolescents: a moderated mediation model
Source: Front Public Health. 2025 Mar 27;13:1502726. doi: 10.3389/fpubh.2025.1502726 (PMC11983463; doi:10.3389/fpubh.2025.1502726)
Supplement: Supplementary file 1 [file Table_1.docx]

The Supplementary Table

The Supplementary Table 1. Depression mediated the relationship between school bullying and internet addiction

| Path |  | B | S.E. | t-statistic | p-value |
| --- | --- | --- | --- | --- | --- |
| Direct |  |  |  |  |  |
| School bullying | internet addiction | 1.53 | 0.007 | 127.4 | ＜0.0001 |
| Indirect |  |  |  |  |  |
| School bullying | internet addiction | 0.56 | 0.020 | 27.8 | ＜0.0001 |
| School bullying | depression | 0.85 | 0.007 | 127.4 | ＜0.0001 |
| depression | internet addiction | 1.14 | 0.009 | 118.7 | ＜0.0001 |

Note: B: regression coefficient; S.E.: standard error.

The Supplementary Table 2. Test for mediation (Depression was used as a mediating variable)

|  | Effect size | S.E. | 95%CI (lower) | 95%CI (upper) | Effect ratio (%) |
| --- | --- | --- | --- | --- | --- |
| Total effect | 1.53 | 0.019 | 1.49 | 1.57 | 100 |
| Direct effect | 0.56 | 0.020 | 0.52 | 0.59 | 36.6 |
| Indirect effect | 0.97 | 0.015 | 0.95 | 1.00 | 63.4 |

Note: S.E.: standard error; CI: confidence interval.

The Supplementary Table 3. Anxiety mediated the relationship between school bullying and internet addiction

| Path |  | B | S.E. | t-statistic | p-value |
| --- | --- | --- | --- | --- | --- |
| Direct |  |  |  |  |  |
| School bullying | internet addiction | 1.53 | 0.020 | 77.0 | ＜0.0001 |
| Indirect |  |  |  |  |  |
| School bullying | Anxiety | 0.69 | 0.006 | 126.7 | ＜0.0001 |
| School bullying | Internet addiction | 0.66 | 0.020 | 32.1 | ＜0.0001 |
| Anxiety | Internet addiction | 1.27 | 0.012 | 105.8 | ＜0.0001 |

Note: B: regression coefficient; S.E.: standard error.

The Supplementary Table 4. Test for mediation (Anxiety was used as a mediating variable)

|  | Effect size | S.E. | 95%CI (lower) | 95%CI (upper) | Effect ratio (%) |
| --- | --- | --- | --- | --- | --- |
| Total effect | 1.53 | 0.020 | 1.49 | 0.157 | 100 |
| Direct effect | 0.65 | 0.020 | 0.62 | 0.70 | 42.5 |
| Indirect effect | 0.88 | 0.014 | 0.85 | 0.90 | 57.5 |

Note: S.E.: standard error; CI: confidence interval.

The Supplementary Table 5. Moderating effect of stigma of mental illness on the relationship between school bullying and internet addiction (Depression was used as a mediating variable)

|  | Internet addiction | Depression |
| --- | --- | --- |
|  | B S.E t-statistic p-value | B S.E t-statistic p-value |
| School bullying | 0.404 0.072 4.20 ＜0.0001 | 0.558 0.024 23.2 ＜0.0001 |
| Depression | 0.889 0.038 23.3 ＜0.0001 |  |
| Stigma of mental illness | -0.139 0.015 -9.4 ＜0.0001 | -0.014 0.005 -2.6 ＜0.01 |
| School bullying×Stigma of mental illness | 0.007 0.002 3.4 ＜0.0001 | 0.008 0.0007 12.4 ＜0.0001 |
| Depression×Stigma of mental illness | 0.008 0.001 6.8 ＜0.0001 |  |
| *R^2^* | 0.207 | 0.172 |
| *F* | 4258.213 ＜0.0001 | 5614.303 ＜0.0001 |

Note: B: regression coefficient; S.E: standard error.

The Supplementary Table 6. Moderating effect of stigma of mental illness on the relationship between school bullying and internet addiction (Anxiety was used as a mediating variable)

|  | Internet addiction | Anxiety |
| --- | --- | --- |
|  | B S.E. t-statistic p-value | B S.E. t-statistic p-value |
| School bullying | 0.354 0.074 4.80 ＜0.0001 | 0.465 0.020 23.7 ＜0.0001 |
| Anxiety | 0.950 0.049 19.4 ＜0.0001 |  |
| Stigma of mental illness | -0.140 0.015 -9.3 ＜0.0001 | -0.010 0.004 -2.1 ＜0.05 |
| School bullying×Stigma of mental illness | 0.008 0.002 4.0 ＜0.0001 | 0.006 0.0005 11.7 ＜0.0001 |
| Anxiety×Stigma of mental illness | 0.010 0.001 6.8 ＜0.0001 |  |
| *R^2^* | 0.183 | 0.170 |
| *F* | 3637.877 ＜0.0001 | 5548.559 ＜0.0001 |

Note: B: regression coefficient; S.E: standard error.
